# ﻿Uneven species occurrence and richness of lowland snakes (Serpentes, Squamata) in Terengganu, Peninsular Malaysia, with new locality records

**DOI:** 10.3897/zookeys.1168.95833

**Published:** 2023-06-26

**Authors:** Muhamad Fatihah Syafiq, Baizul Hafsyam Badli-Sham, Larry Lee Grismer, Amirrudin B. Ahmad

**Affiliations:** 1 Faculty of Science and Marine Environment, Universiti Malaysia Terengganu, 21030, Kuala Nerus, Terengganu, Malaysia; 2 Herpetology Laboratory, Department of Biology, La Sierra University, Riverwalk Parkway, Riverside, California 92505, USA; 3 Institute of Tropical Biodiversity and Sustainable Development, Universiti Malaysia Terengganu, 21030, Kuala Nerus, Terengganu, Malaysia

**Keywords:** Monsoon, reptiles, species richness estimation, species turnover, tropical rainforest

## Abstract

This study documents information on the composition, diversity, richness, and temporal occurrence of snakes at Sekayu’s lowland forest (SLF), Terengganu, Peninsular Malaysia for the first time. The snakes recorded within the SLF were sampled opportunistically from 2013 to 2019, employing the Visual Encounter Survey method (VES) and L-shape pitfall traps with drift fences. Forty-six snake species from 37 genera belonging to the nine families were recorded, of which 11 were new records to Terengganu. Individual-based rarefaction and extrapolation curves were not reaching asymptote, indicating that additional species can be recorded at the study area. Non-parametric species richness estimators estimated and produced a range between 51 and 57 species. ACE was the best estimator based on the quantitative evaluation. All species showed some variations of occurrence patterns across months. Fourteen species were only encountered once across the sampling years, and interestingly 11 of them were only detected during the rainy season (late October to January). In general, the number of species richness, abundance, and rare species were high during this season. Species richness of snakes is high at SLF but sampling effort should be intensified, especially during these rainy months, to obtain a robust estimated snake species richness in SLF. Terengganu harbor considerably high species richness of snakes with a total of 71 species to date (excluding marine snakes), but snake diversity is still underestimated as only a few localities were surveyed in the past years, primarily at the northern part. Future surveys should be commenced at the central and southern parts of Terengganu to complement the current investigation.

## ﻿Introduction

Snakes are some of the most significant faunal components of an ecosystem. They play a crucial role in predator-prey relationships (Marshall et al. 2020; D’souza et al. 2021; Natusch et al. 2021), are highly potential bio-indicators for the ecosystem including for climate change (Bickford et al. 2010; Weatherhead et al. 2012; Böhm et al. 2016; Lourenço-de-Moraes et al. 2019; Zipkin et al. 2020) and habitat degradation monitoring (Todd and Andrews 2008; Beaupre and Douglas 2009; Pike et al. 2010; Shelton et al. 2020). Regrettably, snakes as well as lizards receive poor conservation attention compared to other reptile groups such as tortoises and turtles (Böhm et al. 2013; Shahirah-Ibrahim et al. 2018) and crocodiles and gharials (Martin 2008; Somaweera et al. 2019, 2020).

Malaysia is a tropical region with high endemism and richness of snakes (Roll et al. 2017). Currently, there are at least 191 species reported from Malaysia (MyBis 2021). Although snake species richness in this region is high, the distribution and genetic information are scarce due to limited sampling opportunities (Quah et al. 2018a, b, c; Chan and Grismer 2021a). This is likely because snakes are elusive fauna and notoriously difficult to sample due to their mobility (Barnes et al. 2017; Marshall et al. 2019; Fujishima et al. 2021; Jones et al. 2022), phenological idiosyncrasies (Brown and Shine 2002; Rahman et al. 2013), cryptic morphology and detection, and naturally occur in low densities (Chan and Ahmad 2009; Durso et al. 2011). Therefore, this hampers the conservation efforts and ecological studies of Malaysian snakes (Chan and Grismer 2021a, b).

Terengganu’s forests are still relatively understudied regarding snake diversity compared to other group of reptiles, and most of the information available for snakes are only from herpetofauna checklists, derived from short-term inventories (e.g., Dring 1979; Grismer et al. 2011; Sumarli et al. 2015; Badli-Sham et al. 2019; Zakaria et al. 2019; Fatihah-Syafiq et al. 2020; Komaruddin et al. 2020). In comparison to the lizards, freshwater turtles, and tortoises, ecological studies solely focusing on snakes in Terengganu is non-existent (Grismer and Chan 2008; Grismer et al. 2009, 2014a; Chan and Norhayati 2010; Chan and Chen 2011; Shahirah-Ibrahim et al. 2018).

Sekayu’s lowland forest (SLF), provide a potential site for conducting an ecological study on snake assemblages. A large part of this area resides within the Hulu Terengganu Tambahan Forest Reserve. The lowland forest includes a protected area, Sekayu Recreational Forest (SRF) and Sekayu Agricultural Park (SAP). The latter was developed for agro-based tourism and ecotourism purposes (Bhuiyan et al. 2012). The presence of visitors at these areas may induce human-wildlife conflict, between human and snakes. However, data on snake species richness in SLF is limited. Data to inform park’ managers to spread the awareness and information among the visitors are lacking. Only two snake species, *Najasumatrana* Muller, 1887 and *Tropidolaemuswagleri* (Boie, 1827) are known from this locality based on Zakaria et al. (2019) checklist. Regrettably, this number is likely seriously underestimating the species richness of snakes from this area due to the short-term inventory executed by the study. In contrast, a few new discoveries in various faunal groups were made here such as crabs (Ng and Ahmad 2016; Ng 2020), frogs (Chan et al. 2020), and skinks (Grismer et al. 2014b, 2018; Sumarli et al. 2016), implying that the diversity of fauna is high in this underexplored forest.

Herein, we compiled a checklist of snakes from Sekayu’s lowland forest (SLF) from our study and included information on snakes from previous studies at other localities in Terengganu. We also examined the snake composition using our data at SLF, Terengganu, delivering information about snakes’ diversity, richness, and their temporal occurrence from this locality.

## ﻿Materials and methods

### ﻿Survey area

**Figure 1.**
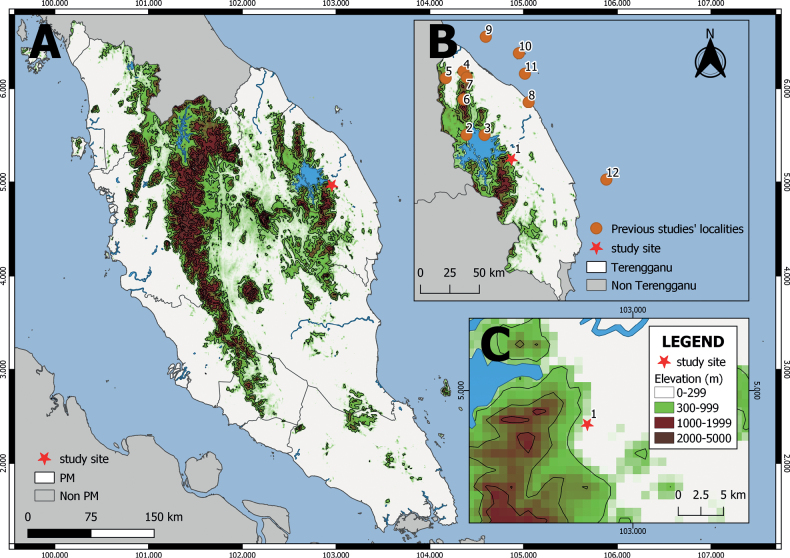
**A** map of Peninsular Malaysia (left) showing the Sekayu’s lowland forest, and the study site indicated by the red star. Insets illustrate **B** the localities of previous studies in Terengganu state where data of snake occurrences were compiled: 1. Sekayu lowland forest, 2. Tembat Forest Reserve, 3. Kenyir Lake, 4. Lata Belatan, 5. Lata Tembakah, 6. Gunung Lawit, 7. Gunung Tebu, 8. Universiti Malaysia Terengganu (UMT), 9. Pulau Perhentian Besar, 10. Pulau Redang, 11. Pulau Bidong, 12. Pulau Tenggol, and **C** the elevation of the study area.

Sekayu’s lowland Forest (**SLF**) is situated in the state of Terengganu, Peninsular Malaysia (4.9676°N, 102.9549°E) (Fig. 1). The Hulu Terengganu Tambahan Forest Reserve comprises approximately 10,899 hectares. The landscape of SLF ranges from flat lowland forest to hilly terrain (area focused upon in this study: ≤ 150 m a.s.l). Primary and secondary dipterocarp forests characterize the vegetation at SLF. The lowland dipterocarp forest can be found at the elevation of < 300 m at the study area, characterized by several tree layers and the upper layers of emergent trees as tall as or > 35 m. The lower strata consist of various species of trees including many small shrubs, herbs, and understory palms (Saw 2010). Common tree species in lowland dipterocarp forests of Hulu Terengganu include *Hopea* spp., *Shorealeprosula*, and *Dipterocarpus* spp. from the major timber family Dipterocarpaceae (Pounsin et al. 2018). Two main streams flow through the study site, namely the Sungai (= river) Bubu and Sungai Peres. These rivers consist of fast-flowing cascades and waterfall at the upper section, followed by rocky and sandy streams in the middle and lower parts. The former river runs through the Sekayu Agricultural Park (**SAP**) and the latter flows through the Sekayu Recreational Forest (**SRF**). Within SAP and SRF, there are anthropogenic structures such as authority offices, roads, and public facilities such as chalets, toilets, cemented walls, wooden huts, as well as including artificial lakes and pools built for various reasons. The mean rainfall of SLF is < 250 mm during the dry period (April to September) and > 250 mm during the rainy period (October to March). The rainy period is also known as monsoon season, and the dry period occurs during the non-monsoon. We divided the non-monsoon season into two categories, post-monsoon (April to August) and pre-monsoon (September) periods. This area has average temperatures of 30 °C and high humidity (> 80%) throughout the year and generally heavy rainfalls are experienced in the months of November and March.

### ﻿Data collection

**Figure 2.**
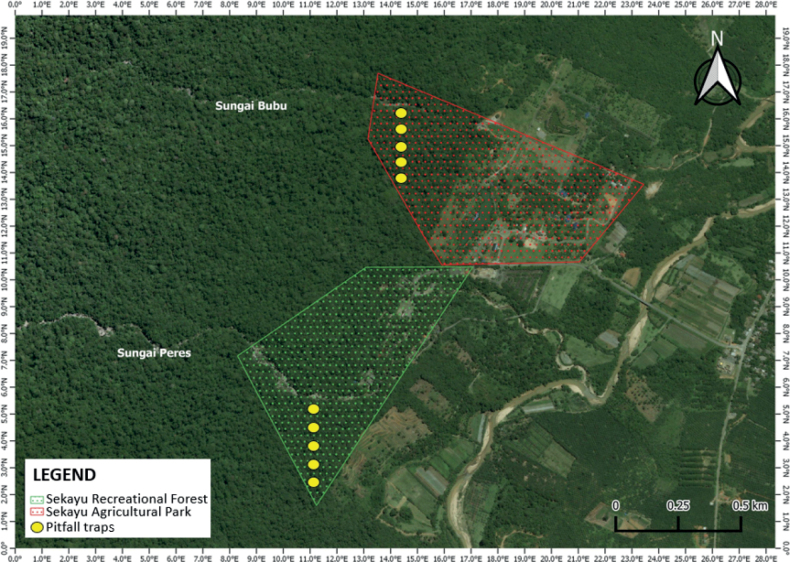
The location of pitfall trap sets at the Sekayu Agricultural Park and Sekayu Recreational Forest in the study area.

Surveys were executed opportunistically during the years 2013–2019. Most of the collections were made for a few months within the year and more surveys were done in the dry period because the SLF was closed between November and February. The collection area spanned the low-lying to the hilly areas (< 300 meters), anthropogenic areas, and along the streams. Visual Encounter Survey (**VES**) and drift-fenced pitfall traps were employed during the study as collecting methods. There were ten sets of pitfall trap installed in the forested area at both Sekayu Recreational Forest and Sekayu Agricultural Park (five sets for each site; Fig. 2). Each set comprised three 18-L buckets with 1.5 m aluminum zinc fences. The pitfalls were set in a straight line. The distance between each set of pitfall traps varied from 20 to 30 m due to geographical constraints. The surveys were aggregated into two time periods: daily surveys (ranging between 1000 hr and 1500 hr) and nocturnal surveys (ranging between 1900 hr and mid-night). Surveys were conducted with field parties consisting of four or five people. Voucher specimens were collected for each species, snakes were euthanized using benzocaine, fixed with 10% formalin, and tagged with the Universiti Malaysia Terengganu Zoological Collection (**UMTZC**) and Universiti Malaysia Terengganu Zoological Collection Photograph (**UMTZCP**) codes before being stored in 70% ethanol (see Appendix 1). Liver tissue was taken before the fixation with formalin and stored in 95% ethanol for future molecular studies. All vouchered specimens were deposited in the General Lab Biology, Universiti Malaysia Terengganu (UMT). Identification of species followed Cox et al. (1998) and Das (2010). The latest taxonomic nomenclature follows The Reptile Database (Uetz et al. 2020). For the consolidated checklist and notes, information was searched for through Google Scholar using English language terms to identify published herpetofaunal studies in Terengganu, in order to obtain available records of the snake species in this state. The following terms in various combinations were used: “herpetofauna”, “reptiles”, “snakes”, and “Terengganu”. Non-peer reviewed sources such as technical reports were excluded.

### ﻿Data analysis

A pie chart, bar chart, and Rank Abundance Curve (RAC) were plotted to assess snake composition and species abundance distribution using Microsoft Excel. The species composition was based on cumulative abundance from all collections since 2013.The Chi-square goodness of fit test was used to fit the species abundance with four abundance models and evaluate which model best fits the dataset of snakes present in Sekayu lowland forests. This test was run using the PAST software (Hammer et al. 2001).

The “iNEXT” R package v. 2.0.20 (Hsieh et al. 2016) was utilized by using R v. 4.1.3 (R Core Team 2022), aided by RStudio integrated development environment (RStudio 2022). The first three Hill numbers (richness, q = 0; Shannon diversity index, q = 1; Simpson diversity index, q = 3) (Hill 1973) were measured. The Hill numbers for these species diversity orders were then used to plot the sample rarefaction and extrapolation curve to measure the sampling effort.

The eight non-parametric species richness estimator values, abundance-based coverage estimator (ACE), incidence-based coverage estimator (ICE), Chao 1 estimator, Chao 2 estimator, first-order Jackknife (Jack 1), second-order Jackknife (Jack 2), Michaelis–Menten Mean (MMMean), and Michaelis–Menten Runs (MMRuns), were calculated using EstimateS v. 9.10 (Colwell 2005). The sample order was randomized 100 times to compute the mean estimator and species richness for each accumulation sample level. To evaluate the estimators, three quantitative evaluation measures were used: bias (scaled mean error), precision (coefficient of variation), and accuracy (scaled mean square error). The bias, precision, and accuracy were calculated following Walther and Moore (2005). Later, each of the measure values for each estimator was ranked accordingly. The value close to “0” was ranked as the number “1” rank and the rank number increased as the estimated value far from “0”. The final ranking was based on a total of each estimator’s number of ranks. The lowest value of the total accumulation was chosen as the best estimator.

The seriation of species presence/absence across months of the sampling years (January to December) was performed using a constrained algorithm (Brower and Kile 1988), done using PAST software. The seriation diagram of species presence/absence was edited to represent species abundance in each respective month. Temporal indices comprised of total turnover, species appearances, and species disappearances were calculated using the “codyn” R package v. 2.0.5 (Collins et al. 2008; Hallett et al. 2016). The total turnover calculated was the proportion of species richness (lost and gained) in relation to the total species in each month-to-month comparison. The turnover metric varied from 0 (no species gained or lost) to 1 (complete species replacement) (Collins et al. 2000).

## ﻿Results

### ﻿Species checklist of snakes in Terengganu

**Figure 3.**
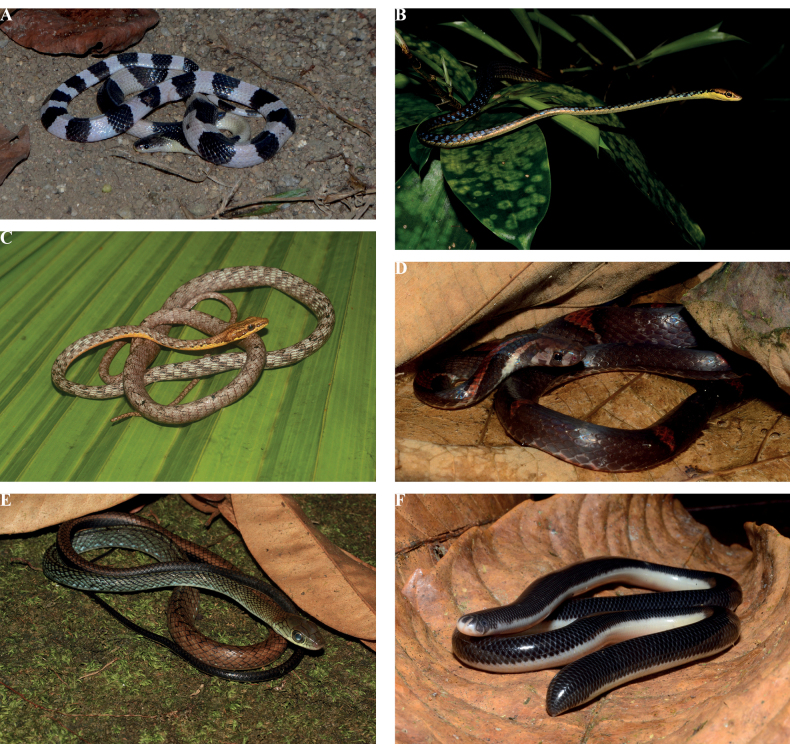
The new records of snakes for Terengganu recorded in SLF**A***Bungaruscandidus***B***Dendrelaphishaasi***C***Dryophiopsrubescens***D***Oligodonsignatus***E***Ptyasfusca***F***Argyrophismuelleri*.

Table 1 incorporates data from this study and previous studies (Grismer et al. 2011; Sumarli et al. 2015; Nur Amalina et al. 2017; Badli-Sham et al. 2019; Zakaria et al. 2019; Fatihah-Syafiq et al. 2020; Komaruddin et al. 2020) that included snake species known to the state of Terengganu to date. This consolidated checklist documents 71 species of snakes found in Terengganu. Of this, 46 snake species from 37 genera belonging to the nine families were recorded from SLF. There were 11 new records acquired from this study, namely *Bungaruscandidus* (Linnaeus, 1758) (Fig. 3A), *Dendrelaphishaasi* Van Rooijen & Vogel, 2008 (Fig. 3B), *Dendrelaphisstriatus* (Cohn, 1905), *Dryophiopsrubescens* (Gray, 1834) (Fig. 3C), *Lycodonalbofuscus* (Dumeril, Bibron & Dumeril, 1854), *Lycodoneffraenis* Cantor, 1847, *Oligodonpurpurascens* (Schlegel, 1837), *Oligodonsignatus* (Gunther, 1864) (Fig. 3D), *Ptyasfusca* (Gunther, 1858) (Fig. 3E), *Argyrophismuelleri* (Schlegel, 1839) (Fig. 3F), and *Xenopeltisunicolor* Reinwardt, 1827 to the state of Terengganu.

**Table 1.** Consolidated checklist of snakes in Terengganu. This list was compiled from results of this study as well as published works of Grismer et al. (2011)^1^, Sumarli et al. (2015)^2^, Nur Amalina et al. (2017)^3^, Badli-Sham et al. (2019)^4^, Zakaria et al. (2019)^5^, Fatihah-Syafiq et al. (2020)^6^, and Komaruddin et al. (2020)^7^. Asterisks (*) denote new records. Codes are included for species recorded from SLF.

No	Code	Family/Species	Pulau Bidong^6^	Pulau Perhentian Besar^1^	Pulau Redang^1^	Pulau Tenggol^1^	Tembat Forest Reserve^3^	Kenyir Lake^5,7^	Lata Belatan^2^	Lata Tembakah^2^	Gunung Lawit^2^	Gunung Tebu^2^	UMT ^4^	Sekayu (this study)
		**Achrochordidae**												
1		*Achrochordusjavanicus* Hornstedt, 1787					x							
		**Colubridae**												
2	Ahmyc	*Ahaetullamycterizans* (Linnaeus, 1758)					x							x
3	Ahpra	*Ahaetullaprasina* (Boie, 1827)		x	x	x		x					x	x
4	Bocyn	*Boigacynodon* (Boie, 1827)					x							x
5	Bodra	*Boigadrapiezii* (Boie, 1827)							x		x	x		x
6	Bojas	*Boigajaspidea* (Dumeril, Bibron & Dumeril, 1854)					x		x					x
7	Bomel	*Boigamelanota* (Boulenger, 1896)		x	x		x	x			x		x	x
8	Bonig	*Boiganigriceps* (Gunther, 1863)									x	x		x
9		*Calamarialumbricoidea* Boie, 1827					x							
10	Capav	*Calamariapavimentata* Dumeril, Bibron & Dumeril, 1854					x	x						x
11	Chorn	*Chrysopeleaornata* (Shaw, 1802)		x			x						x	x
12	Chpar	*Chrysopeleaparadisi* Boie, 1827					x							x
13	Chpel	*Chrysopeleapelias* (Linnaeus, 1758)					x							x
14	Cofla	*Coelognathusflavolineatus* (Schlegel, 1837)					x	x						x
15		*Coelognathusradiatus* (Boie, 1827)											x	
16	Decau	*Dendrelaphiscaudolineatus* (Gray, 1834)					x							x
17		*Dendrelaphiscyanochloris* (Wall, 1921)										x		
18	Defor	*Dendrelaphisformosus* (Boie, 1827)								x				x
19	Dehaa	Dendrelaphishaasi* Van Rooijen & Vogel, 2008												x
20	Depic	*Dendrelaphispictus* (Gmelin, 1789)		x	x		x	x					x	x
21	Destr	Dendrelaphisstriatus* (Cohn, 1905)												x
22	Drrub	Dryophiopsrubescens* (Gray, 1834)												x
23	Lyalb	Lycodonalbofuscus* (Dumeril, Bibron & Dumeril, 1854)												x
24	Lycap	*Lycodoncapucinus* Boie, 1827	x	x										x
25	Lyeff	Lycodoneffraenis* Cantor, 1847												x
26	Lysuba	*Lycodonsubannulatus* (Dumeril, Bibron & Dumeril, 1854)		x										x
27	Lysubc	*Lycodonsubcinctus* Boie, 1827		x			x		x					x
28		*Gonglyosomalongicauda* (Peters, 1871)					x							
29		*Gonyosomaprasinum* (Blyth, 1854)					x							
30	Gooxy	*Gonyosomaoxycephalum* (Boie, 1827)		x										x
31		*Oligodonoctolineatus* (Schneider, 1801)					x							
32	Olpur	Oligodonpurpurascens* (Schlegel, 1837)												x
33	Olsig	Oligodonsignatus* (Gunther, 1864)												x
34	Pslon	*Pseudorhabdionlongiceps* (Canthor,1847)					x							x
35		Pseudorhabdioncf.longiceps										x		
36		*Ptyascarinata* (Gunther, 1858)					x							
37	Ptfus	Ptyasfusca* (Gunther, 1858)												x
38		*Ptyaskorros* (Schlegel, 1837)					x							
39		*Xenelaphishexagonotus* (Cantor, 1847)					x							
40	Xeuni	Xenopeltisunicolor* Reinwardt, 1827												x
		**Elapidae**												
41	Bucan	Bungaruscandidus* (Linnaeus, 1758)												x
42		*Bungarusfasciatus* (Schneider, 1801)					x							
43	Bufla	*Bungarusflaviceps* Reindhart, 1843					x	x						x
44		*Calliophisbivirgatus* (Boie, 1827)					x		x					
45	Caint	*Calliophisintestinalis* (Laurenti, 1768)					x							x
46	Nakou	*Najakaouthia* Lesson, 1831					x						x	x
47	Nasum	*Najasumatrana* Muller, 1887					x							x
48		*Ophiophagushannah* (Cantor, 1836)					x							
		**Homalopsidae**												
49	Enenh	*Enhydrisenhydris* (Schneider, 1799)					x						x	x
50	Hyplu	*Hypsiscopusplumbea* (Boie, 1827)					x		x				x	x
51	Hobuc	*Homalopsisbuccata* (Linnaeus, 1758)					x	x					x	x
52		*Phytolopsispunctata* Gray, 1849					x							
		**Natricidae**												
53		*Rhabdophisflaviceps* (Dumeril, Bibron & Dumeril, 1854					x							
54		*Rhabdophisrhodomelas* (Boie, 1827)								x				
55	Rhchr	*Rhabdophischrysargos* (Schlegel, 1837)					x				x			x
56		*Rhabdophissubminiatus* (Schlegel, 1837)					x							
57	Xetri	*Xenochrophistrianguligerus* (Boie, 1827)					x							x
58		*Fowleapiscator* (Schneider, 1799)					x							
		**Pareidae**												
59	Apboa	*Aplopelturaboa* (Boie, 1828)										x		x
60		*Asthenodipsaslaevis* (Boie, 1827)					x							
61	Pacar	*Pareascarinatus* Wagler, 1830					x							x
62	Pamar	*Pareasmargaritophorus* (Jan, 1866)							x					x
		**Pythonidae**												
63	Maret	*Malayopythonreticulatus* (Schneider, 1801)	x	x	x	x	x						x	x
64		*Pythonbrongersmai* Stull, 1938					x							
		**Typhlophidae**												
65		*Argyrophisdiardii* (Schlegel, 1839)					x							
66	Inbra	*Indotyphlopsbraminus* (Daudin, 1803)	x	x										x
67	Armue	Argyrophismuelleri* (Schlegel, 1839)												x
		**Viperidae**												
68	Trwag	*Tropidolaemuswagleri* (Boie, 1827)		x			x			x				x
69		*Trimeresurushageni* (Lidth De Jeude, 1886)						x		x				
70		*Trimeresurussabahi* Regenass & Kramer, 1981					x				x	x		
71		*Trimeresurussumatranus* (Raffles, 1822)									x			

### ﻿Notes on the new record species and their distribution in Peninsular Malaysia

#### ﻿ Bungarus candidus

Taxon classificationAnimaliaSquamataElapidae

(Linnaeus, 1758)

671390B2-A258-5D01-83F7-1008DF756C5A

Fig. 3A Blue Krait Snake

##### Natural history notes.

This species can be identified by its cylindrical body with enlarged vertebral scale row; head not distinct from neck; head black dorsally and connected with the first body marking forming chevron shape; body with black crossbands with white interspaces; chin, neck, and ventral of the body white. Most individuals were frequently found during the rainy period (October to March), either crossing roads or foraging near the slow-flowing stream.

##### Distribution.

This species is known from a few localities from the states of Kedah, Kelantan, and Johor (Grismer and Pan 2008; Muin et al. 2017; Ayob et al. 2020).

#### ﻿ Dendrelaphis haasi

Taxon classificationAnimaliaSquamataColubridae

Van Rooijen & Vogel, 2008

F50753DD-864D-5744-B18F-2400E05B925A

Fig. 3B Haas’ Bronzeback Snake

##### Natural history notes.

This species can be identified by its slender body; head orangish to pale brown color dorsally; narrow postocular stripe covering less than half of the temporal region, with some black spots at the lower temporal region; and dull ventrolateral stripe. The species was found ~ 1100 hr sleeping on a twig and leaf of an ornamental tree (0.5 m high above the ground) in the plant nursery situated adjacent to the secondary forest.

##### Distribution.

van Rooijen and Vogel (2008) stated that this species is widely distributed in Peninsular Malaysia, but Pulau Tioman was the only locality mentioned in their article. Since then, no subsequent article has reported the occurrence of this species in any other locality. This study reports the first locality record of this species in Peninsular Malaysia, specifically in the state of Terengganu.

#### ﻿ Dendrelaphis striatus

Taxon classificationAnimaliaSquamataColubridae

(Cohn, 1905)

4432FE9D-02E2-5C0E-8767-46CA6EC1832D

##### Natural history notes.

This species can be identified by its slender body; head bronze-brown in color; thick black stripe extending from the snout passing through the eye and ending at the neck region; neck yellow when inflated; body yellow at the anterior and blue at the posterior with oblique black band. The species was found sleeping during night (~ 2100 hr) on the ornamental tree near the Sekayu Recreational Forest authority’s office.

##### Distribution.

This species is widely distributed in Peninsular Malaysia (MyBis 2021) but there is no record of occurrence of this species specifically from Terengganu state in any published documentation to our knowledge.

#### ﻿ Dryophiops rubescens

Taxon classificationAnimaliaSquamataColubridae

(Gray, 1834)

9246786C-C8A6-5C95-8F2C-B09701064503

Fig. 3C Brown Whip Snake

##### Natural history notes.

This species can be identified by its slender but laterally compressed body; head pale greyish brown dorsally with three distinct short brown stripes at the occipital region; thick dark brown stripe extending from snout, through the eye to the nape area; body greyish to brown dorsally with dark brown and cream spots. The species was found sleeping at (~ 2000 hr) on a twig of a dipterocarp tree (2 m height above ground) situated near the stream.

##### Distribution.

This species is widely distributed in Peninsular Malaysia (MyBis 2021) but there is no record of occurrence of this species specifically from Terengganu state to our knowledge.

#### ﻿ Lycodon albofuscus

Taxon classificationAnimaliaSquamataColubridae

(Duméril, Bibron & Duméril, 1854)

60B67FE9-5BC9-50CD-8AA9-B94748BAD453

##### Natural history notes.

This species can be identified by its elongated, slender body; elongated, depressed head; blunt snout; dorsal body uniformly grey in color; pale ventrally; dorsal scale strongly keeled. An individual was found at night between (~ 2100 hr to 2300 hr), crossing the established trail adjacent to the secondary forest, near a fast-flowing stream.

##### Distribution.

This species was previously recorded from a few localities such as Pasoh Forest Reserve, Krau Wildlife Reserve, and Pulau Tioman (MyBis 2021) and the species is now reported from Terengganu state for the first time.

#### ﻿ Lycodon effraenis

Taxon classificationAnimaliaSquamataColubridae

Cantor, 1847

83A6EA26-01F5-5AD0-BA3C-FE6F1EB7252D

##### Natural history notes.

This species can be identified by its slender body, head dark brown with white stripes extending from snout, passing through the eye and ending before the nape; dorsal body dark brown with white irregularly shaped crossbands. The species was found sleeping on the tree vines (2 m height above ground) situated near a slow-flowing rocky stream.

##### Distribution.

The species has been reported from the states of Kelantan, Johor, and Pahang (MyBis 2021). To our knowledge, this species has not been recorded in Terengganu, which located in between Kelantan to the north and Pahang to the south on the northeastern part of Peninsular Malaysia. Hence this finding confirmed the presence of this species in Terengganu state.

#### ﻿ Oligodon purpurascens

Taxon classificationAnimaliaSquamataColubridae

(Schlegel, 1837)

FDAF39D0-AF77-5547-8B9D-1768530DDA4F

##### Natural history notes.

This species can be identified by its robust body; head dark purplish with brown ocular bars; dorsal body dark brown with faint blotches and irregular crossbands. The species was observed at night (~ 2200 hr) on the ground near a slow-flowing stream.

##### Distribution.

This species is widely distributed in Peninsular Malaysia (MyBis 2021).

#### ﻿ Oligodon signatus

Taxon classificationAnimaliaSquamataColubridae

(Günther, 1864)

C7E3AEA1-D01E-5268-A935-7BE96565E399

Fig. 3D Barred Kukri Snake

##### Natural history notes.

This species can be identified by its robust body; head pale brown dorsally with dark brown ocular bars; dorsal body dark brown with reddish brown triangular markings; first red crossbar had a chevron pattern pointing towards the head. The species was found on the leaf litter substrate near the slow-flowing stream.

##### Distribution.

Based on Chan and Ahmad (2009), this rare species was reported to occur in the states of Selangor, Melaka, Johor, Pahang, and Negeri Sembilan. From the previous reports (e.g., Tweedie 1954; Hendrickson 1966), this species was only recorded in the southern part of Peninsular Malaysia. The findings of this study extended its distribution range to the northwestern part of Peninsular Malaysia, with a new locality.

#### ﻿ Ptyas fusca

Taxon classificationAnimaliaSquamataColubridae

(Günther, 1858)

3242B040-E42A-5F1E-BB80-9B89C964E380

Fig. 3E White-bellied Rat Snake

##### Natural history notes.

This species can be identified by its olive-green body dorsally and white ventral surface; black stripes present at the sides of the posterior body and tail. The species was found sleeping on a twig (3 m height above the ground) during a rainy night at about 2100 hr.

##### Distribution.

This species was reported from Pahang and Johor (MyBis 2021). In the IUCN Red List of Threatened Species, this species is reported to be widely distributed in Peninsular Malaysia, Sumatra, Borneo, and southern Thailand, south of the Isthmus of Kra. As far as we know, this new record extends its distribution range further north in the northeastern part of Peninsular Malaysia.

#### ﻿ Argyrophis muelleri

Taxon classificationAnimaliaSquamataTyphlopidae

(Schlegel, 1839)

6B79BE48-97D3-5EB5-9872-663947B141ED

Fig. 3F Müller’s Blind Snake

##### Natural history notes.

This species can be identified by its cylindrical body; head black dorsally; head indistinct from the neck; vestigial eyes; black dorsum; white ventrally; tail with sharp, terminal spine. The species was found foraging at night around 2200 hr in a man-made drain.

##### Distribution.

This species was reported from Perak, Pahang, and Johor (MyBis 2021). Despite being a widely distributed species, this species has never been reported from the state of Terengganu, and the distribution in Peninsular Malaysia is now extended to the northeastern part with a new record.

#### ﻿ Xenopeltis unicolor

Taxon classificationAnimaliaSquamataXenopeltidae

Reinwardt, 1827

3F3BFF93-64A5-5893-A8CC-13E911C68557

##### Natural history notes.

This species can be identified by its relatively robust body; body and head brown in color dorsally but producing an iridescence under strong light; white ventrally; body scale smooth. The species was found foraging at night between 2000 hr and 2300 hr on the ground (sandy substrate) near the large fast-flowing stream.

##### Distribution.

This species was reported from Kedah, Pulau Pinang, Negeri Sembilan, and Pahang (MyBis 2021). Despite being a widely distributed species, this species has never been reported from the state of Terengganu, and the distribution in Peninsular Malaysia is now extended to the northeast.

### ﻿Species abundance distribution and composition

**Figure 4.**
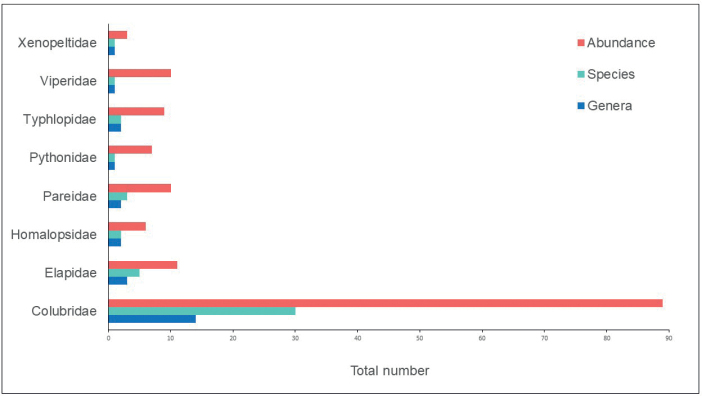
Each snake family’s total abundance, species, and genera inhabiting Sekayu Lowland Forest.

The family Colubridae (89 individuals) has the highest number of individuals recorded in the study area (Fig. 4), followed by Elapidae (11 individuals), Paridae, Viperidae (ten individuals), Typhlophidae (nine individuals), Pythonidae (seven individuals), Homalopsidae (six individuals), and Xenopeltidae (three individuals). Genera-wise, the family has the highest number of genera (14 genera), followed by Elapidae (three genera), Homalopsidae, Paridae, Typhlophidae (two genera), and the remainder of the family with only one genus each. Concerning species richness, the family Colubridae was the most species-rich taxon (30 species), followed by Elapidae (five species), Paridae (three species), Homalopsidae, and Typhlophidae (two species), and the rest of the family with one species each (Table 1).

**Figure 5.**
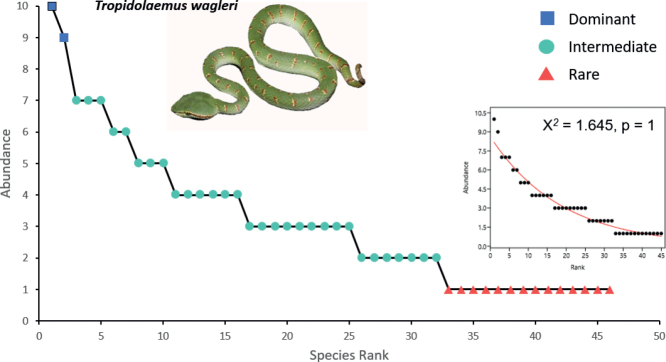
Rank abundance curve for snakes in Sekayu Lowland Forest. The X-axis indicates species rank, while the Y-axis denotes the numerical abundance of each species. Species were ranked from the most abundant to the rare species. The blue rectangle represents dominant species, the green circle represents intermediate species, and the red triangle represents rare species. The inset curve was the best fitted geometric series (Χ*^2^* = 1.645) model curve obtained from PAST software while the photograph is of the most abundant snake species, *Tropidolaemuswagleri*, recorded in this study.

Fig. 5 showed that there are two dominant species, with *Tropidolaemuswagleri* had the highest number of individuals (ten individuals). Seven species were doubletons. Singletons in the rank abundance curve hereafter were considered as rare species recorded in this study. Thirteen rare species were recorded, while the remainder were intermediately abundant species. The species abundance distribution of snakes in SLF best fitted the geometric series model (Χ^*2*^ = 1.65).

### ﻿Sampling effort and species richness estimation

**Figure 6.**
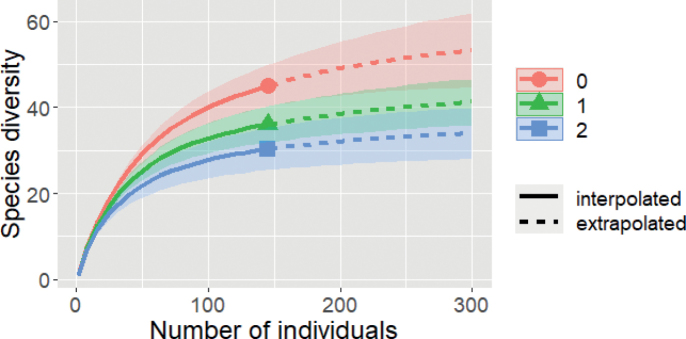
Individual-based rarefaction (solid line segment) and extrapolation (dashed line segment) sampling curves with 95% confidence intervals (shaded areas) for diversity orders: q = 0 (species richness), q = 1 (Shannon’s diversity), q = 2 (Simpson’s diversity).

Individual-based rarefaction and extrapolation curves demonstrated that the curve for diversity measures of species richness (q = 0) does not reach the asymptote, even after the sample size was doubled to 300 individuals by the extrapolation (Fig. 6). The curves for diversity measures of Shannon’s diversity (q = 1) and Simpson’s diversity (q = 2) also showed an inclining trend that was not stabilized even when the sample size was increased and extrapolated. Having said that, the Simpson’s diversity (q = 2) was superficially approaching asymptotic with the increasing abundance.

Table 2 showed that the estimated values from the non-parametric species richness estimators were varied between 51 and 73 species. An additional 5–27 species were expected by the non-parametric species richness estimators from the observed species richness. The least-bias estimator was MMMeans. All estimators seem to be highly precise except the two coverage-based estimators, ICE and ACE. However, ACE shows the most accurate species richness estimator in this study while MMRuns was the least accurate. Based on the final ranking, ACE was chosen as the best estimator to estimate the species richness of snakes in SLF, while the MMRuns estimator had the worst performance.

**Table 2.** Estimated values from eight non-parametric species richness estimators with their evaluation measures: bias, precision, and accuracy. Value 0 indicates no bias, high precision, and high accuracy. The ranking of the eight non-parametric estimators was based on their performance of each measure. The final ranking for each estimator was measured based on the summation of their performance (= total rank accumulation). Sobs = Observed species richness.

Estimators	Estimated value	Bias	Precision	Accuracy	Total Rank	Final Rank
Sobs (*n* = 46)						
ACE	52.48	-0.14 (2)	0.13 (2)	0.02 (1)	5	1
Chao 1	54.68	-0.20 (4)	0 (1)	0.05 (2)	7	2
MMMeans	66.79	0.05 (1)	0 (1)	0.16 (6)	8	3
Jack 2	60.04	-0.19 (3)	0 (1)	0.15 (5)	9	4
Chao 2	51.13	-0.24 (6)	0 (1)	0.10 (3)	10	5
Jack 1	57.25	-0.22 (5)	0 (1)	0.11 (4)	10	5
ICE	54.45	0.19 (3)	1.84 (3)	0.64 (7)	13	6
MMRuns	73.19	1.50 (7)	0 (1)	9.40 (8)	16	7

### ﻿Temporal occurrence of snakes across the sampling years

**Figure 7.**
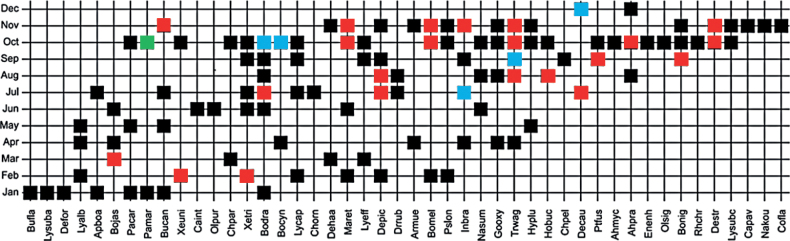
The seriation diagram of the species abundance in respective months over the sampling years (2013–2019). The X-axis indicates species name, and the Y-axis indicates months. Note: black rectangle = one individual, red rectangle = two individuals, blue rectangle = three individuals, green rectangle = four individuals. See Table 1 for species codes.

The data of 46 species of snakes recorded at SLF showed that more snake species were observed in October (26 species) while December had the lowest number of two species observed (Fig. 7). *Pareascarinatus* was the most frequently detected species in October (four individuals). The species that were detected thrice in the respective month across the sampling years were: July = *I.braminus*; September = *T.wagleri*; October = *B.cynodon* and *B.drapiezii*; December = *D.caudolineatus*. The species that were detected twice in the respective month across the sampling years were: February = *X.trianguligerus* and *X.unicolor*; March = *B.jaspidea*; July = *B.drapiezii*, *D.caudolineatus*, and *D.pictus*; August = *D.pictus*, *H.buccata*, and *T.wagleri*; September = *B.nigriceps* and *P.fusca*; October = *A.prasina*, *B.melanota*, *D.striatus*, *M.reticulatus*, and *T.wagleri*; November = *B.melanota*, *B.candidus* (Fig. 3A), *D.striatus*, *I.braminus*, *M.reticulatus*, and *T.wagleri*. The remaining species occurred only once throughout the months across the sampling years. In general, many species were recorded during early monsoon months (October–November) but fewer during the monsoon (December–January). However, the data showed that singletons/unique observations (black square) of snakes were not restricted in either monsoon (e.g, January) or non-monsoon (April–June) months.

Fig. 7 also shows that many species that were found during Jan–Feb and Feb–Mar were also found in other months during the dry and pre-rainy periods, namely *Aplopelturaboa* (Jan and Jul), *Boigadrapiezii* (Jan, Jun, Jul, Aug, Sep, and Oct), *Boigajaspidea* (Mar, Apr, and Jun), *Boigamelanota* (Feb, Oct and Nov), *Bungaruscandidus* (Jan, May, Jul and Nov), *Chrysopeleaparadisi* (Mar and October), *Dendrelaphishaasi* (March and November), *Dendrelaphispictus* (February, July, August, Sep and Nov), *Lycodonalbofuscus* (Feb, Apr and May), *Lycodoncapucinus* (Feb, Jul, Sep and Oct), *Lycodoneffraenis* (Mar, Sep and Oct), *Malayopythonreticulatus* (Feb, Jun, Oct, and Nov), *Pareascarinatus* (Jan, May and Oct), *Pareasmargaritophorus* (Jan and Oct), *Pseudorhabdionlongiceps* (Feb, Oct and Nov), *Xenochrophistrianguligerus* (Jan, Jun, July, Sep, and Oct), and *Xenopeltisunicolor* (Feb and Oct).

The months of May-June were in the middle of the dry period. The species found in May were *Bungaruscandidus*, *Hypsiscopusplumbea*, *Lycodonalbofuscus*, and *Pareascarinatus*. Meanwhile, the species found in June were *Boigadrapiezii*, *Boigajaspidea*, *Calliophisintestinalis*, *Malayopythonreticulatus*, *Najasumatrana*, *Oligodonpurpurascens*, and *Xenochrophistrianguligerus*. Both months showed a different observed species, indicating complete species replacement from May to June. The same pattern of differences with the different composition of snake species was also observed between the months Nov–Dec. This explained the high value of the turnover index for both pairs of months, May-Jun, and Nov–Dec. In addition, no species was found consistently every month across the 12 months of the sampling years.

**Figure 8.**
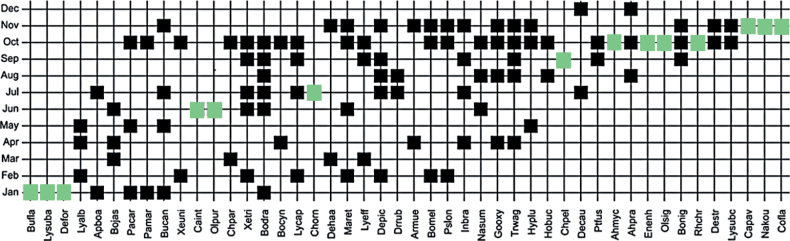
The seriation diagram of the species presents in respective months over the sampling years. The X-axis indicates species name, Y-axis indicates months. Note: Pale green rectangle = only one individual found across the months over the sampling years. See Table 1 for species codes.

Based on the presence/absence data, rare species that occurred only once (unique species) across the sampling years were detected more frequently during the pre-monsoon (October) and monsoon months (November and January) (Fig. 8). Unique species were reported during dry season as well (June and July). Species such as *Bungarusflaviceps*, *Dendrelaphisformosus*, and *Lycodonsubannulatus* were detected only in January while *Calliophisintestinalis* and *Oligodonpurpurascens* were seen once in June and *Chyropeleaornata* was detected in July. *Chrysopeleapelias* was detected in September only. In October, four unique species were recorded, namely *Ahaetullamycterizans*, *Enhydrisenhydris*, *Oligodonsignatus*, and *Rhabdophischrysargos* and in November, *Calamariapavimentata*, *Coelognathusflavolineatus*, and *Najakaouthia* were recorded.

**Figure 9.**
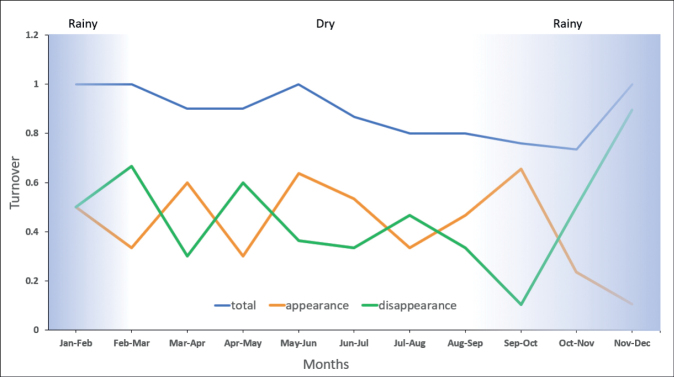
The turnover plot depicts the cumulative month-to-month total turnover with species appearances and disappearances. The blue shaded area indicates the rainy season, while the non-shaded area indicates the dry season.

Turnover index values varied over time in this study (Fig. 9). Species appearance was the highest and species disappearance was the lowest between September-October. Species appearance dropped drastically starting from the month September-October to November-December coinciding with the monsoon season. On the contrary, species disappearance rocketed from September-October to November-December as the monsoon season arrived.

## ﻿Discussion

This study elevates the current knowledge of snakes in Terengganu regarding new records, species richness, and temporal occurrence of species at Sekayu’s lowland forest (SLF). The number of snake species found in SLF represents 64% of the total recorded snake species found in Terengganu (Table 1). To date, SLF is regarded as the locality with the highest species richness of snakes in Terengganu. Tembat Forest Reserve, a site comparable with SLF due to the deployment of similar methods and efforts, had two species fewer than SLF, for which Nur Amalina et al. (2017) obtained a total of 44 species. Despite that, while we observed 46 species of snakes in SLF, we did not observe 16 of the species that were documented by Nur Amalina et al. (2017). In contrast, their study did not record 22 species that were recorded in our study. In total, 65 species of snakes were documented from both sites. The marked difference in species composition of snakes between both studies warrants future studies that compare the reptile diversity (snakes in particular) between these sites to elucidate the species distribution and diversity pattern and intersite similarity of the two locations.

Eleven new records were obtained in this study. These species are widespread and distributed around Peninsular Malaysia (MyBis 2021). However, these species were not found previously in other localities in Terengganu including the offshore islands (Table 1). In general, snakes are naturally rare and elusive due to their cryptic morphology, phenological idiosyncrasies, and climate and habitat-sensitivity, causing low detectability of snake species during inventories (Durso et al. 2011; Ward et al. 2017; Frazao et al. 2020; Asad et al. 2021). This factor may explain why these 11 species were previously not observed in other localities in Terengganu’s forested area.

Two species ranked as the topmost abundant snakes in SLF, *Tropidolaemuswagleri* (ten individuals) and *Boigadrapiezii* (nine individuals). The widespread distribution and many occurrence records (Vogel et al. 2007; HerpMapper 2023) of the former species may indicate that the species is more readily detected. Despite that, natural history information such as prey items and movement of most of the recorded snakes, including these two topmost abundant species, is limited to ascertain and explain their occurrence at the study area. However, multiple records at other sites in Terengganu (Table 1) imply that *T.wagleri* can inhabit a wide range of habitats including offshore islands while *B.drapiezii* can be found in most lowland to hilly areas near water bodies such as streams, which is the geographical characteristic of the study area.

The low number of dominant species (two species) with large proportions of rare species (14 singletons) results in high unevenness of SLF’s snake assemblage (Fig. 5). The geometric series model was chosen as the best model to describe species abundance distribution for the snake assemblage of SLF. The large proportions of the singletons resulted from the rarity and elusiveness of these 14 species clearly shaped the species distribution pattern. These species only occurred once across the sampling years (Fig. 8). Despite being sampled relatively well, these species were really difficult to spot and are highly elusive species.

The individual-based rarefaction and extrapolation curves demonstrate that the sampling of snake species in SLF is not yet complete (Fig. 6). The species diversities showed an increasing trend that was not yet stabilized. Hence, the observed species richness from this study may not represent the true species richness of snakes in SLF. This may also be true for the abundance and evenness of the snake assemblages. This study attempted to estimate the species richness of snakes in SLF. Based on Table 2, an additional 5–27 species could be discovered with continuous sampling in the future. However, some of these estimates could be over-estimated due to the biasness, precision, and accuracy of the estimators used. This study evaluated the utilized estimators and found that the ACE estimator performed the best among the estimators (Table 2). The ACE estimator was moderately precise and had relatively low biasness (Table 2). The performance of low biasness and high precision of the ACE estimator in this study is also shown by the performance of this estimator in the study by Hortal et al. (2006). According to that study, the ACE estimator’s advantage is that it is non-sensitive to the grain sizes (sampling effort units). Snake richness and abundance in the snake inventories can vary due to different methods used, sites, and times (e.g., Sumarli et al. 2015; Nur Amalina et al. 2017; Fiorillo et al. 2021). Hence, an estimator with such an advantage is crucial to estimate the snake species richness. According to this estimator, 54 snakes were estimated to be discovered at SLF, adding six species to the observed species richness in this study.

A study by Kery (2002) in Europe demonstrated that the probability of finding snake species might vary depending on habitat, year, season, the area surveyed, the population size of the species, and the observer. Hence, the occurrence of the snake species in the respective months in this study may or may not also apply to the same snake assemblages in other localities in Terengganu. However, we provided essential information on which months the respective snake species can be detected in this study. For instance, some species were repeatedly found in the same month over the sampling years (Fig. 7).

The snake species richness was the highest in Oct (Fig. 7). Consequently, species appearances were the highest during the transition of Sep to Oct (Fig. 9). The month of Sep marked the beginning of the monsoon season. Asad et al. (2021) demonstrated that snake species occurrence in Borneo was positively associated with humidity and rainfall. Although we did not statistically test the relationship between the rainfall and the snake assemblage, we postulate that the increase of humidity and the rainy period in Oct might also influence species richness and abundance (Figs 7, 9). Some of the species were found twice to four times during this month over the sampling years (Fig. 7): their breeding phenology may explain their high numbers of occurrences at certain months (Cox et al. 1998; Das 2010). Additionally, the snake species’ high occurrence may also coincide with increased prey activity during the rainy period (Brown and Shine 2002; Natusch et al. 2021) and signal the onset of hunting period for the snake species (Natusch et al. 2022). This interaction has been demonstrated by previous studies on scrub python, *Simaliaamethistina* (Schneider, 1801) in Australia, that has seasonal prey items (Natusch et al. 2021; 2022). Another example was a study in Thailand that radio-tracked *Boigacyanea* (Dumeril, Bibron & Dumeril, 1854), and found an increase in their movements, space use, and activity during the nesting season of its prey, a songbird (D’souza et al. 2021). These results indicate that snakes have seasonal prey items, and their increased activity may be associated with prey nesting and the wet season. The previous studies used radio-tracking and yielded novel insights into the natural history, movement, and behavioral ecology of their snake species (D’souza et al. 2021; Natusch et al. 2021, 2022); hence this method also should be applied to the snakes recorded in this study area in the future to gain such information.

The heavy rain might interrupt the visual of the search parties hence causing low species richness detected in these months. Overall, these results elucidate that effort to sample snake species in SLF could be maximized during these rainy months to improve snake detection. Three other rare species, *Calliophisintestinalis*, *Chrysopeleaornata*, and *Oligodonpurpurascens*, were found during the dry period. Sperry and Weatherhead (2008) found that the loss of and shifts in water availability increased the activity of the terrestrial snake *Pantherophisobosletus* (Say, 1823) in the USA. This might also be the case for the occurrence of terrestrial species of the first and last species above. Terrestrial snakes may prefer to be close to the riparian areas during the dry period (Asad et al. 2021), hence increasing the detectability of these species at our sampling sites (Figs 7, 9). Study that investigates and correlate snake species richness and abundance with rainfall and sampling sites (i.e., riparian versus hilly areas) in Terengganu should be conducted to test this hypothesis. We hope the information from our study can stimulate such study.

Herpetofaunal studies in riparian forests in Peninsular Malaysia have demonstrated that this forest type harbor significant number of species richness not only limited to reptiles but also amphibians, with new records and species (e.g., Chan et al. 2020; Badli-Sham et al. 2021; Fatihah-Syafiq et al. 2021; Quah et al. 2021). Previous studies suggested that riparian habitats should be preserved to reduce the extinction risk of many snake species as this habitat support high species richness (Todd et al. 2017; Guzy et al. 2019). Todd et al. (2017) discovered that human-dominated landscapes exacerbated snake species richness for those species that consume small vertebrates and species associated with aquatic habitats, and that species with these traits occurred more frequently in a natural landscape. Many of the observed species in Sekayu Lowland Forest (SLF) have similar traits, thus explaining the high species richness of snakes in this area. This number reflects the need for sustainable management of SLF particularly of the remaining undisturbed habitats of this area to safeguard the snake species.

## ﻿Conclusions

Despite the SLF location within the forest reserve, many of the riparian forests within such reserves in Terengganu have been transformed into anthropogenic recreational areas (e.g., Lata Belatan and Lata Tembakah). This is worrying because unsustainable development and other anthropogenic activities affect reptile species richness, particularly of snakes (Gillespie et al. 2015; Bauder et al. 2020; Doherty et al. 2020; Mohd Izam et al. 2021). Sekayu’s lowland forest has become the major source of new reptile species discoveries in Terengganu (Grismer et al. 2014b, 2018; Sumarli et al. 2016), implying that the remaining intact forests in SLF and other riparian forested areas in Terengganu should be preserved so that their yet unknown species are not lost before they are officially described (e.g., Grismer et al. 2016; Nur Amalina et al. 2017). The fact that SLF has two frequented localities by local visitors (Bhuiyan et al. 2012) increases the possibility of human-wildlife conflict between human and snakes. We hope the information available from this study is used to inform to park’s authorities in SLF to spread awareness among the visitors to reduce such human-wildlife conflict. Overall, the results of this study echo the SLF’s paramount importance as a potential conservation area for snakes of the Terengganu.

## Supplementary Material

XML Treatment for Bungarus candidus

XML Treatment for Dendrelaphis haasi

XML Treatment for Dendrelaphis striatus

XML Treatment for Dryophiops rubescens

XML Treatment for Lycodon albofuscus

XML Treatment for Lycodon effraenis

XML Treatment for Oligodon purpurascens

XML Treatment for Oligodon signatus

XML Treatment for Ptyas fusca

XML Treatment for Argyrophis muelleri

XML Treatment for Xenopeltis unicolor

## ﻿Additional information

### Author ORCIDs

Muhamad Fatihah Syafiq https://orcid.org/0000-0002-1185-3653

Baizul Hafsyam Badli-Sham https://orcid.org/0000-0003-2106-3361

Larry Lee Grismer https://orcid.org/0000-0001-8422-3698

Amirrudin B. Ahmad https://orcid.org/0000-0002-7775-1289

### Data availability

All of the data that support the findings of this study are available in the main text or Supplementary Information.

## ﻿Appendix 1

**Table A1.** List of species with UMTZC code.

No.	Species name	Code
1	*Ahaetullamycterizans*	UMTZCP021113-2367
2	*Ahaetullaprasina*	UMTZC1354
3	*Boigacynodon*	UMTZCP141120-7424
4	*Boigadrapiezii*	Specimen not preserved
5	*Boigajaspidea*	Specimen not preserved
6	*Boigamelanota*	UMTZC1651
7	*Boiganigriceps*	Specimen not preserved
8	*Calamariapavimentata*	UMTZC1477
9	*Chrysopeleaornata*	UMTZC1639
10	*Chrysopeleaparadisi*	Specimen not preserved
11	*Chrysopeleapelias*	Specimen not preserved
12	*Coelognathusflavolineatus*	Specimen not preserved
13	*Dendrelaphiscaudolineatus*	UMTZC1364
14	*Dendrelaphisformosus*	Specimen not preserved
15	*Dendrelaphishaasi*	Specimen not preserved
16	*Dendrelaphispictus*	UMTZCP270214-4490
17	*Dendrelaphisstriatus*	UMTZCP021113-2393
18	*Dryophiopsrubescens*	UMTZC1808
19	*Lycodonalbofuscus*	UMTZC1771
20	*Lycodoncapucinus*	UMTZC1071
21	*Lycodoneffraenis*	UMTZC1618
22	*Lycodonsubannulatus*	UMTZCP280214-4580
23	*Lycodonsubcinctus*	UMTZC1658
24	*Gonyosomaoxycephalum*	UMTZC1676
25	*Oligodonpurpurascens*	Specimen not preserved
26	*Oligodonsignatus*	Specimen not preserved
27	*Pseudorhabdionlongiceps*	UMTZCP170422-647
28	*Ptyasfusca*	UMTZC1807
29	*Xenopeltisunicolor*	UMTZC1638
30	*Bungaruscandidus*	UMTZC1697
31	*Bungarusflaviceps*	UMTZC1698
32	*Calliophisintestinalis*	UMTZC1770
33	*Najakaouthia*	UMTZC1660
34	*Najasumatrana*	UMTZC1550
35	*Enhydrisenhydris*	UMTZC1648
36	*Hypsiscopusplumbea*	UMTZC1655
37	*Homalopsisbuccata*	UMTZC1677
38	*Rhabdophischrysargos*	Specimen not preserved
39	*Xenochrophistrianguligerus*	UMTZC1038
40	*Aplopelturaboa*	UMTZCP070214-4228
41	*Pareascarinatus*	UMTZC1356
42	*Pareasmargaritophorus*	UMTZC1667
43	*Malayopythonreticulatus*	Specimen not preserved
44	*Indotyphlopsbraminus*	UMTZC1476
45	*Argyrophismuelleri*	UMTZC1636
46	*Tropidolaemuswagleri*	UMTZC1652
